# Three-dimensional speckle-tracking echocardiography offers complete volumetric and functional assessment of the left atrium

**DOI:** 10.1007/s10554-021-02220-4

**Published:** 2021-03-30

**Authors:** Attila Nemes

**Affiliations:** Department of Medicine, Medical Faculty, Albert Szent-Györgyi Clinical Center, University of Szeged, Semmelweis Street 8, P.O. Box 427, Szeged, 6725 Hungary

I am reading the paper of Sanoglioni et al. aiming to to identify to evaluate the usefulness of left atrial (LA) reservoir strain for risk stratification of asymptomatic patients with moderate aortic stenosis [1]. In recent years, newer echocardiographic techniques, including two-dimensional (2D) speckle-tracking echocardiography (STE) and three-dimensional echocardiography (3D) came into practice offering detailed insights into not only volumetric, but functional changes of heart chambers during the cardiac cycle including the left atrium (LA) [2]. 3DSTE merges all advantages of 3D echocardiography and STE: the heart can be seen as a 3D organ, and due to speckle-tracking algorithm, quantification of deformation of wall motion by numeric parameters is also possible using virtually created 3D cast of the heart chambers [2]. Moreover, not only volumetric changes respecting the cardiac cycle could be detected by 3DSTE, but contractility of wall segments could be quantified by global, regional and segmental strain, rotational and dyssynchrony variables. Another important advantage of the method is that all these parameters could be calculated from the same digitally acquired 3D echocardiographic pyramidal dataset (‘echocloud’) at the same time making the whole analysis non-invasive, easy-to-perform and fast with a relatively short learning curve [2]. Although 3DSTE has been introduced within a relatively short period and more time is needed for it to be routinely used in the clinical practice, normal reference values of LA volumetric and strain parameters are available now. Several studies validated 3DSTE-derived LA parameters against that assessed by two-dimensional echocardiography, 2DSTE, real-time 3D echocardiography and computer tomography [2]. It has also been demonstrated that in certain disorders, specific volumetric and functional alterations of the LA may be present represented by complex 3DSTE-derived parameters [2]. However, significant importance of the software used for strain analysis has been emphasized in literature [2]. Hopefully, 3DSTE will become one of the most important non-invasive imaging techniques in the future among others with its ability of detailed analysis of the LA.
